# Phytochemical nanoencapsulation and microfluidics drive gene and tumor microenvironment modulation

**DOI:** 10.3389/fphar.2025.1694752

**Published:** 2025-09-29

**Authors:** Ana Belen Peñaherrera-Pazmiño, Mishell Criollo, Rebeca Gonzalez-Pastor

**Affiliations:** Centro de Investigación Biomédica (CENBIO), Facultad de Ciencias de la Salud Eugenio Espejo, Universidad UTE, Quito, Ecuador

**Keywords:** phytochemical nanosystems, cancer nanotherapy, tumor microenvironment, gene modulation, microfluidics, microphysiological systems, translational nanomedicine, SDG 3

## Abstract

Phytochemicals are plant-derived bioactive compounds with promising anticancer properties, but their clinical use is limited by poor solubility, instability, rapid metabolism, and restricted tumor penetration. Nanoencapsulation strategies address these barriers by enhancing bioavailability, stability, and tissue-specific delivery, thereby improving therapeutic efficacy and reducing systemic toxicity. This mini-review summarizes recent progress in nanoscale phytochemical delivery systems engineered for gene modulation and tumor microenvironment targeting, including lipid-based, polymeric, hybrid, and biogenic nanocarriers that improve biodistribution and enhance cellular uptake. Notably, the functional performance of nanoscale delivery systems depends on precisely controlled physicochemical characteristics. Consequently, microfluidics has emerged as a powerful tool to fine-tune and fabricate phytochemical-based nanocarriers in a reproducible manner. Beyond fabrication, microfluidic lab-on-a-chip platforms recreate physiological and tumor-specific microenvironments, providing dynamic, real-time assessment of drug transport, metabolism, and tumor–vascular interactions in biomimetic conditions that surpass conventional static models. These innovations expand mechanistic understanding and support more predictive preclinical evaluations. Remaining challenges include variability of natural sources, limited pharmacokinetic and toxicological data, and hurdles in scale-up and standardization. By integrating nanoscale engineering with microfluidic innovation, phytochemical-based nanomedicine is positioned to advance toward more effective, safer, and clinically translatable cancer therapies.

## 1 Introduction

Phytochemicals, a diverse group of bioactive compounds found in plants, are the subject of renewed interest in biomedical research for their chemopreventive and chemotherapeutic properties (George et al., 2021; Subramaniam et al., 2019). Despite the widespread use of synthetic pharmaceuticals (Campos et al., 2019; Tamatam and Mohammed, 2024), these natural bioactives have long been valued in traditional medicine and are increasingly recognized for their multi-targeted biological mechanisms and generally favorable safety profiles (Hoenders et al., 2024; Wink, 2022; Yang and Ling, 2025). Their anticancer potential has been supported by preclinical studies and, in some cases, by clinical evidence (Choudhari et al., 2019). However, the transition of phytochemicals from bench to bedside remains challenging due to factors such as low water solubility, chemical instability, limited tissue penetration, and rapid metabolism, all of which restrict bioavailability and therapeutic impact *in vivo*.

To overcome these barriers, advanced formulation strategies have been developed to enhance solubility, protect functional integrity, and achieve controlled, site-specific delivery (Chen et al., 2024) to support clinical translation (Aljabali et al., 2025). Among these, encapsulation strategies for phytochemicals encompass both micro- and nanoscale systems. Microencapsulation techniques, such as spray-drying and freeze-drying, are commonly employed to protect bioactive molecules from environmental degradation and to improve handling, shelf stability, and palatability (Ijod et al., 2024; Bińkowska et al., 2024; Ingale et al., 2025). Some are engineered to respond to physiological cues, such as pH or enzymatic activity, which allow for site-specific release of bioactive compounds or secondary delivery systems that mediate therapeutic effects (Ang et al., 2019; Ma Y. et al., 2021). However, their role in targeted gene or microenvironmental modulation remains limited. In contrast, nanoscale delivery systems, including liposomes, polymeric nanoparticles, and exosome-based carriers, are specifically designed to interact with biological systems, providing control over biodistribution, cellular uptake, and enhanced therapeutic performance (Das et al., 2020). Their functional performance depends on precisely controlled physicochemical characteristics, requiring reproducible, scalable fabrication (Ly et al., 2024; Hui et al., 2025).

Traditional batch-based synthesis often suffers from limitations such as batch-to-batch variability and reduced control over particle uniformity and surface characteristics (Mülhopt et al., 2018). In contrast, microfluidic technologies enable precise, reproducible fabrication of phytochemical nanocarriers by controlling formulation parameters such as flow rate, concentration, and mixing dynamics. This approach produces uniform nanoparticles with defined surface and compositional properties, supporting scalable and translational nanomedicine development (Bezelya et al., 2023; Sebastian, 2022). Beyond synthesis, microfluidic technologies support functional evaluation through biomimetic platforms replicating tissue-specific microenvironments with greater fidelity than conventional 2D or static 3D cultures. These systems offer dynamic control over nutrient delivery, waste removal, and oxygen gradients under flow (Kim et al., 2021; Ayuso et al., 2022). In cancer research, they allow reconstruction of complex tumor architectures, incorporating vasculature, stromal barriers, hypoxic zones, and even microbiota (Ayuso et al., 2022; Farooqi et al., 2023; Zhai et al., 2024). Their efficiency with minimal cell input makes them ideal for patient-derived material, while precise flow control supports real-time assessment of metastasis, drug response, and tumor–vascular niche interactions (Farooqi et al., 2023; Ngo et al., 2023; Zhai et al., 2024).

**FIGURE 1:**
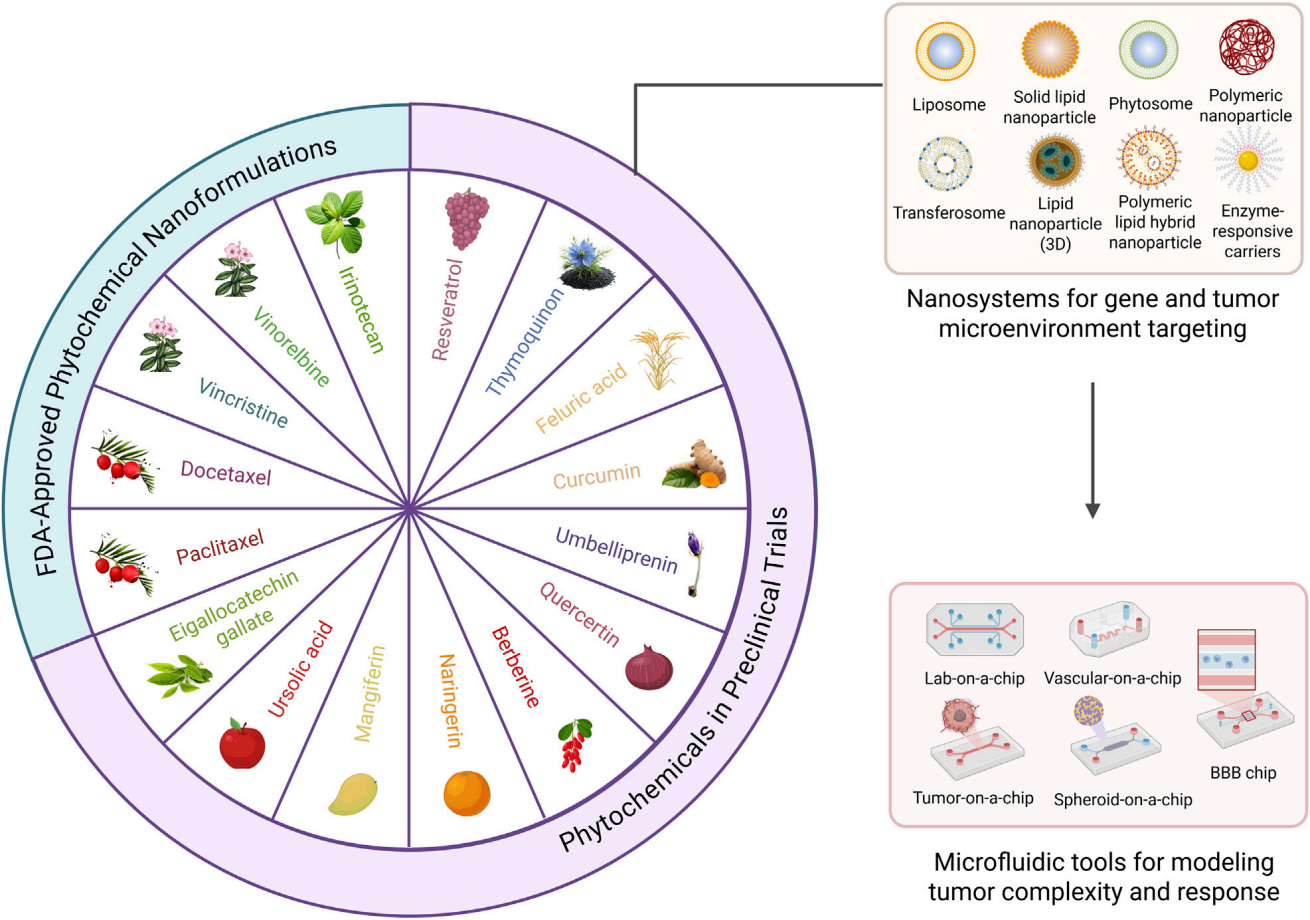
Phytochemicals used in oncology, their sources, and their delivery and evaluation systems.

This minireview focuses on nanoscale phytochemical delivery systems, highlighting microfluidic-based synthesis and functional evaluation (Figure 1), with emphasis on gene modulation and tumor microenvironment targeting.

## 2 Phytochemical-based nanosystems for gene and tumor microenvironment targeting

### 2.1 Formulation challenges and delivery strategies

While plant-derived drugs such as vincristine, paclitaxel, and docetaxel are established in oncology, they represent only a small fraction of phytochemicals with demonstrated preclinical anticancer activity that have yet to translate into routine clinical use (Figure 1) (Asma et al., 2022; Mazumder et al., 2022; Kim et al., 2024). The majority of plant-derived agents exhibit poor solubility and bioavailability, requiring higher doses that increase toxicity risk. Assumption of inherent safety is common (Jităreanu et al., 2022; Mugale et al., 2024), yet many plant-based compounds, especially alkaloids, saponins, and anthraquinones, can cause organ-specific toxicity and genotoxicity, may alter drug metabolism, and exacerbate adverse effects (Quan et al., 2020; Rao et al., 2022; Brewer and Chen, 2017). Consequently, establishing no-observed-adverse-effect levels (NOAEL) (Dorato and Engelhardt, 2005) through standardized toxicokinetic studies is essential for clinical advancement (Al-Naqeb et al., 2024). These challenges have driven the development of nanoscale delivery strategies that enhance pharmacokinetics and formulation versatility by solubilizing poorly water-soluble compounds, protecting them from enzymatic degradation, extending circulation time, and ultimately improving bioavailability while reducing toxicity (Dhupal and Chowdhury, 2020; Bilia et al., 2019).

Liposomes remain a cornerstone in phytochemical nanoformulation due to their biocompatibility and amphiphilic nature. Liposomal formulations of resveratrol and epigallocatechin gallate improved bioavailability and reduced systemic toxicity in colorectal and liver cancer models (Wilson et al., 2023). Other lipid-based systems (Jacob et al., 2025), such as solid lipid nanoparticles and nanostructured lipid carriers, enhance absorption and plasma retention of betulinic acid and andrographolide in various tumor models (Wang et al., 2024; Li H. et al., 2022). Phytosomes (Talebi et al., 2025), formed by complexing phytochemicals with phospholipids, improve membrane permeability and metabolic stability, particularly benefiting poorly absorbed polyphenols, silymarin, and catechin (Babazadeh et al., 2018). In contrast to phytosomes, polymeric and hybrid nanocarriers offer versatility for structurally diverse phytochemicals. Systems based in poly(lactic-co-glycolic acid) (PLGA) and hybrid carriers like lecithin–chitosan, often combined with polyethylene glycol (PEG)-modified surfactants, further enhance solubility, systemic retention, and cellular uptake of compounds such as epicatechin, quercetin, ursolic acid, thymoquinone, naringenin, and berberine in various cancer models (Perez-Ruiz et al., 2018; Wang et al., 2021; Shivani et al., 2023; Hsu et al., 2024; Selmi et al., 2023; Bhia et al., 2021). Beyond improving solubility, nanosystems can also overcome structural barriers to reach tumors more effectively. In melanoma models, transferosomes, ultradeformable liposomes that can squeeze through narrow intercellular gaps (Mohaddish et al., 2025), enabled increased skin penetration and boosted antitumor activity of flavonoid-rich extracts (Motawea et al., 2024). Additionally, incorporating matrix-modulating agents such as hyaluronidase further enhances intratumoral penetration of quercetin and resveratrol (Sun et al., 2025; Yu et al., 2024).

Controlled-release strategies exploit tumor-specific cues to trigger release, maintaining therapeutic levels while reducing systemic toxicity and dosing (Park et al., 2021). Chemical-responsive systems use acid-labile or disulfide linkages to destabilize in the acidic, glutathione-rich tumor milieu. Examples include quercetin in pH-responsive graphene-oxide/polymer nanocarriers, epigallocatechin-3-gallate in pH-sensitive nanoparticles, and ursolic acid delivered either in chitosan-modified liposomes (pH-responsive) or as a redox-responsive polymeric prodrug, all showing enhanced intracellular release under tumor-mimicking conditions (Matiyani et al., 2022; Bhattacharya et al., 2024; Wang et al., 2017; Fu et al., 2021). Enzyme-responsive carriers, such as matrix metalloproteinases (MMP)-cleavable block copolymers, exploit selective cleavage by tumor-overexpressed proteases (Vizovisek et al., 2021), achieving faster release than non-cleavable controls (Padmavathy et al., 2018). Similarly, dendrimers and hydrogels can be engineered for the progressive degradation of their structural networks via proteolysis to sustain polyphenol release and improve dispersibility (Ben-Zichri et al., 2022), and localized delivery of silibinin while minimizing burst-related toxicity (Yin et al., 2023). Targeted nanodelivery augments passive accumulation by decorating carriers with ligands for tumor-overexpressed receptors or organ-specific uptake, thereby boosting in-tumor exposure of phytochemicals while sparing normal tissues (Kalia and Kaur, 2020). Collectively, strategies such as folate, RGD (Arg-Gly-Asp) peptide, apolipoprotein E (ApoE), galactose, and epithelial cell adhesion molecule (EpCAM) have been applied to resveratrol, quercetin, epigallocatechin-3-gallate, curcumin, betulinic acid, paclitaxel, and others across diverse tumor types, in some cases also eliciting anti-tumorigenic immune responses (Barbarisi et al., 2018; Kazi et al., 2020; Dutta et al., 2024; Chen et al., 2022; Hu et al., 2021). These have enabled delivery to hard-to-reach tumors such as gliomas (via transferrin, lactoferrin, or sialic acid-mediated blood–brain barrier (BBB) transport) and to cancer stem cell niches through CD44-targeted systems (Guo et al., 2013; Yang et al., 2017; Kuo et al., 2019; Mittal et al., 2023). These ligand-mediated approaches expand the range of tumors accessible to phytochemical-based nanotherapy, including those protected by physiological barriers or with low passive accumulation.

Among these strategies, curcumin, a benchmark compound, has been incorporated into nearly all major nanocarrier platforms, exemplifying design principles that can be applied to other phytochemicals (Zandieh et al., 2023). Similarly, while whole plant extracts remain under exploration, batch variability complicates standardization and regulation compared to isolated compounds (Ukwubile et al., 2025; Alabrahim et al., 2024; Guillén-Meléndez et al., 2024). In addition, plant-derived exosome-like nanoparticles have emerged as biogenic delivery platforms that naturally encapsulate and transport phytochemicals across biological barriers, offering high biocompatibility, therapeutic potential, targeting capability, and efficient cellular uptake (Kim et al., 2022; Sha et al., 2024).

### 2.2 Mechanistic insights and clinical translation

Phytochemicals delivered through nanocarriers produce antiproliferative and pro-apoptotic effects by modulating core oncogenic cascades as well as oxidative-stress sensing via Kelch-like ECH-associated protein 1–nuclear factor erythroid 2–related factor 2 (Keap1–Nrf2) (Parvin et al., 2025; Aljabali et al., 2025; Situmorang et al., 2024). Certain subclasses display mechanistic preferences: polyphenols like resveratrol and curcumin frequently downregulate nuclear factor kappa B (NF-κB) and signal transducer and activator of transcription 3 (STAT3), impairing inflammatory and survival cascades (Dhupal and Chowdhury, 2020); flavonoids including quercetin and epigallocatechin gallate modulate phosphatidylinositol 3-kinase (PI3K)/protein kinase B (Akt)/mammalian target of rapamycin (mTOR) and mitogen-activated protein kinase/extracellular signal-regulated kinase (MAPK/ERK) signaling, restricting proliferation and metabolic adaptation (Melim et al., 2022); terpenoids such as ursolic acid and oridonin directly interfere with STAT3, heat shock protein 70 (HSP70), and other protein effectors (Yao et al., 2023); and alkaloids like berberine predominantly target AMP-activated protein kinase (AMPK)-dependent metabolic reprogramming, indirectly suppressing mTOR-driven growth (Hashim et al., 2024; Cheng and Ji, 2020). In parallel, phytochemicals also alter glycolysis, glutamine dependence, and lipid biosynthesis, reducing tumor metabolic plasticity (Dey et al., 2021; Hashim et al., 2024; Shuvalov et al., 2023; Wu et al., 2021). Epigenetically, phytochemicals, notably polyphenols and terpenoids, can alter histone acetylation and DNA methylation, as well as modulate miRNA expression, restoring silenced tumor suppressor genes and reducing oncogene expression, yielding antiproliferative, pro-apoptotic, and resensitizing effects (Aljabali et al., 2025; Dandawate et al., 2016; Roy and Datta, 2019). These molecular-level interventions converge to restrict both bulk tumor populations and therapy-persistent subclones, including cancer stem-like cells (Dandawate et al., 2016; Melim et al., 2022; Naujokat and McKee, 2021).

Beyond intrinsic signaling, nanocarrier-delivered phytochemicals modulate the tumor microenvironment (TME) by regulating fibroblast activation, extracellular matrix remodeling, angiogenesis, and immune cell function (Melim et al., 2022; Aljabali et al., 2025). Polyphenols blunt epithelial-to-mesenchymal transition (EMT)–metastasis programs and hypoxia-inducible factor 1α/vascular endothelial growth factor (HIF-1α/VEGF)-driven angiogenesis, flavonoids modulate stemness pathways such as Wingless/Integrated (Wnt)/β-catenin, Hedgehog, and Notch, and terpenoids engage apoptosis and p53-centered DNA-damage checkpoints (Situmorang et al., 2024; Aljabali et al., 2025; Liu et al., 2023; Ci et al., 2016; Kim et al., 2024; Pan et al., 2025; Shuvalov et al., 2023). Importantly, phytochemicals enhance antigen presentation and cytotoxic T-cell infiltration and modulate immunosuppressive mediators such as programmed death-ligand 1 (PD-L1) and transforming growth factor-β (TGF-β) in a compound- and context-dependent manner (Parvin et al., 2025; Dhupal and Chowdhury, 2020). They also promote macrophage polarization from an M2-like to an M1-like phenotype and suppress the accumulation of myeloid-derived suppressor cells, thereby restoring antitumor immune surveillance (Yao et al., 2023; Aljabali et al., 2025). Resveratrol, curcumin, and catechins synergize with checkpoint inhibitors by reprogramming the immunosuppressive microenvironment, while ursolic acid and quercetin modulate inflammatory factors to shift TME balance toward tumor rejection (Chen et al., 2020; Guven et al., 2022; Li et al., 2023b). Combination nanoformulations, widely explored for curcumin, resveratrol, quercetin, and other phytochemicals, have enabled the co-delivery of chemotherapeutics or radiotherapy to achieve synergistic antitumor effects (Kang et al., 2018; Cheng and Ji, 2020; Lv et al., 2016; Wongrakpanich et al., 2024; Afereydoon et al., 2022; AbouAitah et al., 2022; Afshari et al., 2023; Corte-Real et al., 2024), counteracting multidrug resistance by downregulating efflux transporters and disrupting pro-survival metabolic adaptations (Parvin et al., 2025; Melim et al., 2022; Li et al., 2019). More recently, nanocarriers have been engineered to co-deliver therapeutic genes, such as p53, regulatory RNAs (siRNA and miRNA targeting PD-L1, survivin, VEGF, B-cell lymphoma 2 (Bcl-2), and others), and naturally occurring cytolytic peptides, thereby enhancing therapeutic efficacy through gene silencing, apoptosis induction, and immune modulation (Ashrafizadeh et al., 2020; Bhagavatheeswaran and Balakrishnan, 2023; Li X. et al., 2022; Eksi et al., 2025; Wang et al., 2025; Xu et al., 2018; Sun et al., 2023). Collectively, these combination approaches leverage multiple signaling and metabolic pathways, reprogram the tumor microenvironment, and mitigate therapeutic resistance.

Several phytochemical nanoformulations, such as paclitaxel (Abraxane^®^), irinotecan (Onivyde^®^), vinorelbine (NanoVNB^®^), vincristine (Marqibo^®^), and docetaxel (DoceAqualip^®^), are already FDA-approved for cancer therapy (Dhupal and Chowdhury, 2020), while multiple curcumin-, camptothecin-, ursolic acid-, mangiferin-, and quercetin-based platforms are undergoing clinical evaluation in liposomal, polymeric, micellar, and plant-derived nanocarriers (Devan et al., 2023; Lekhak and Bhattarai, 2024; Kumar et al., 2024). Patent activity highlights curcumin-, resveratrol-, and quercetin-based nanosystems, such as liposomes, polymeric nanoparticles, and plant-derived vesicles, designed for improved solubility, stability, and tumor targeting (Table 1). Translation remains limited by pharmacokinetic, scale-up, and regulatory hurdles; standardized characterization and robust preclinical models and clinical design will be critical for bringing these promising nanosystems from bench to bedside.

**TABLE 1:** Advances in phytochemical-based nanosystems for cancer therapy: patent trends and microfluidic production approaches.

Part A. Patent activity in phytochemical-based nanosystems for cancer therapy (2010–2022)					
Active ID	Phytochemical(s)	Nanoplatform	Purpose	Indications(s)	Year
WO2010013224A2 (Pending)	Curcumin	Liposomal formulation	Enhance bioavailability and targeted delivery	Cancer (general)	2010
US20200188311A1 (Active)	Curcumin (with STAT3 inhibitor and a chemotherapeutic agent)	Plant/derived microvesicle	Cancer targeting moiety	Brain, breast, lung, and colon cancer	2017
WO2017137957A1 (Active)	Resveratrol	Colloidally stable nanoparticles	Improve bioavailability and half-life	Cancer, cardiovascular disorders	2017
US20170224636A1 (Inactive)	Curcumin-sophorolipid complex	Nanoemulsion	Improve solubility, stability, and oral bioavailability	Breast cancer	2017
EP3144006 (Active)	Curcumin	Liposomes	Combined with chemotherapeutics, eliminates QT prolongation	Glioblastoma	2017
US10182997B2 (Active)	Curcumin	Polymeric nanoparticles	Enhance solubility and therapeutic effect	Cancer (general)	2018
WO2018098247A1 (Active)	Broccoli extract	Plant-derived nanoparticles	Improve anticancer potential	Colon cancer	2018
IN202141046188 (Active)	Quercetin	TPGS nanosuspension	Improve dissolution and oral bioavailability	Breast cancer	2021
IN202241000705 (Pending)	Astragalus (with cisplatin and vinorelbine)	Nanoformulation	Enhance anticancer potential	Lung cancer	2022
US12268785B2 (Active)	Curcumin	Nano/micro particles	Enhanced stability and bioavailability	Cancer, inflammation	2022

Part B. Summary of recent studies on the production of nanocarriers for phytochemical encapsulation using microfluidic devices								
Nanocarrier	Microfluidic technique	Size (nm)	PDI	EE (%)	LC (%)	Composition	Embedded phytochemical	Ref
Polymeric nanoparticles	flow-focus microfluidic chip	76.5 ± 0.8	<0.3	97.2 ± 0.6	11.1 ± 0.1	zein-SH nanoparticle suspension	Curcumin	Guo et al. (2023)
Hybrid nanoparticles (HNPs)	multi-stage microfluidic TrH chip	131.4 ± 1.32 143.2 ± 2.25	<0.3	≈100	14.97 ± 1.19 16.58 ± 0.69	(PTX)-simvastatin/HNPs, PTX-lenvatinib//HNPs	Paclitaxel	Li et al. (2025)
Liposomes	SHM	∼120	<0.2	N.A.	17	DMPC/curcumin	Curcumin	Hamano et al. (2019)
Liposomes	5-input chip mixing junction, Dolomite	227 ± 1	0.20 ± 0.01	38.0 ± 6.0	11.0 ± 2.0	DSPC:Chol PEG2000-PE 1:2 doxorubicin:umbelliprenin	Umbelliprenin	Gkionis et al. (2020)
Liposomes	MHF	147 ± 19	0.124	88	6.5	DMPC/Chol/PTX	Paclitaxel	Jaradat et al. (2022)
Liposomes	MHF	168 ± 4.5	0.183	91	6.8	DPPC/Cholesterol/PTX	Paclitaxel	Jaradat et al. (2022)
Liposomes	NanoAssemblr^®^ Benchtop herringbone	79.3 ± 7.3	0.125 ± 0.057	83 ± 5	N.A.	oleous phase: Chol, PC, Mal-PEG- docetaxel aqueous phase: dextrose, salt buffer (MgSO_4_ or KH2PO_4_)	Docetaxel	Dacos et al. (2024)

## 3 Microfluidic technologies driving innovation in design and evaluation

### 3.1 Microfluidic synthesis of phytochemical nanocarriers: precision and scalability

One of the unique properties of microfluidics is laminar flow, an ordered parallel flow devoid of any fluid layer disruption, which confers constant continuous mixing through the process (Jaradat et al., 2022). In this profile, diffusion plays a key role in evening out concentration differences at a molecular level (Cullen and Misra, 2015). Microfluidics creates steep spatial and temporal solubility gradients, critical for uniform, well-defined nanoparticles (Nunziata et al., 2025). These properties confer a superior mixing quality, assure the same production quality over time with minimal intra-batch, reduced batch-to-batch, and operator variability during scale-up (Cai et al., 2022).

Nanoparticle synthesis comprises three stages: nucleation, growth, and particle separation (Siavashy et al., 2021). Batch nanoparticle synthesis methods lack control in particle growth, mixing, and separation to prevent agglomeration, ultimately causing fluctuations in size distribution and a diverse particle assortment with varying chemical and physical traits, which restricts the synthesis of core–shell nanoparticles and diminishes encapsulation efficiencies (EE). On the other hand, microfluidics enables precise flow rate control (Cai et al., 2022). Then, it can overcome some of the large-scale reactor intricate hurdles as it tackles variability and scalability issues, which are major conventional batch technique concerns (Nunziata et al., 2025). In the nanocarrier synthesis realm, these advantages have been exploited to control attributes such as size, size distribution, and drug loading (Table 1).

Regarding polymeric nanoparticles, chitosan is an FDA-recognized biopolymer cleared for use in wound-healing devices and with limited Generally Recognized As Safe (GRAS) status in specific food applications, also widely investigated for drug delivery systems (Shah et al., 2025; Naghib et al., 2024). Beyond its prior use in nanomedicine, it has more recently been integrated into microfluidic platforms for the controlled synthesis of nanoparticles, underscoring its translational relevance (Siavashy et al., 2021). More broadly, microfluidic approaches have enabled the synthesis of other polymeric nanoparticles as well. For example, using the innovative multi-stage microfluidic TrH chip, hybrid nanoparticles co-encapsulating paclitaxel-simvastatin and paclitaxel-lenvatinib were successfully produced, demonstrating the versatility of microfluidic platforms for multi-drug-loaded nanomedicines (Li et al., 2025).

Regarding liposome preparation, microhydrodynamic focusing (MHF) (Weaver et al., 2022) and herringbone micromixer (Pisani et al., 2022) have been utilized. MHF presents promising results in producing liposomal formulations with low polydispersity index (PDI) by a one-step procedure (Bochicchio et al., 2020). In MHF, two fluid streams are introduced into a microchannel, where one stream flows at the center and is enveloped by another. This mixture relies on the diffusion mechanism to blend two reagents, wrestling in a lower throughput of around a hundred μL/min. In contrast, herringbone micromixers boast a higher throughput of approximately a few mL/min. Most studies utilizing this configuration rely on a commercialized chip that requires specialized equipment, the NanoAssemblr™ bench-top instrument (Precision Nano Systems Inc., Vancouver, Canada). This device is designed for nanocarrier production and is commercially accessible for research endeavors. In fact, the characteristics of eleven liposomal docetaxel formulations prepared using the NanoAssemblr™ bench-top instrument were assessed by (Dacos et al., 2024). Indeed, the liposomal delivery system for curcumin (Lipo-Cur) was developed utilizing automated microfluidics. When administered alongside cisplatin to mice with tumors, Lipo-Cur strengthened the cisplatin antitumor effectiveness across various mouse tumor models while mitigating nephrotoxicity (Hamano et al., 2019). In this connection, umbelliprenin was co-encapsulated with doxorubicin in liposomes, and this combination, prepared with microfluidics, induced higher toxicity than liposomes prepared with the thin-film method, with an IC_50_ (half-maximal inhibitory concentration) at least 2-fold lower. This feature was attributed to different release kinetics. Furthermore, they discovered that umbelliprenin affected the viscoelastic behaviour and the lipid biomembrane fluidity (Gkionis et al., 2020).

In this regard, (Jaradat et al., 2022), prepared liposomes to encapsulate paclitaxel and determined the best lipid candidates for nanocarrier synthesis. They found that microfluidics has a significant effect in improving the EE of paclitaxel compared to other conventional methods, such as film hydration and extrusion (EE% <50%). Furthermore, they observed that MHF enhanced 1,2-Dimyristoyl-*sn*-*glycero*-3-phosphocholine (DMPC) and 1,2-Dipalmitoyl-*sn*-*glycero*-3-phosphocholine (DPPC) EE%. In addition, these lipids provided a smaller particle size due to their short acyl length. Besides, paclitaxel loading in both DMPC and DPPC liposomes exhibits higher packing with DPPC and shows a sustained release profile. Importantly, the delayed release (after 24 h) can be an advantage in limiting the collateral toxicity to normal tissue due to the reduced premature release.

In fact, continuous flow achieved through microfluidics provides better heat mass transport, and it enables multiple unit operations (Guo et al., 2023). These properties were applied to successfully load curcumin, a hydrophobic polyphenol extracted from the rhizomes of *Curcuma longa,* into zein-SH nanoparticles by establishing a robust and controllable solvent-antisolvent laminar diffusion, achieving a millisecond short mixing time and a homogeneous particle size distribution. They improved EE and loading capacity (LC) (Table 1, Part B) by applying microfluidics when compared to bulk mixing prepared nanoparticles (EE% = 7.7 ± 0.5, LC% = 0.4 ± 0.5). They found that at a high flow ratio, the nucleated nanoparticles are rapidly diluted in the antisolvent and kinetically locked, preventing further size growth. When prepared by bulk mixing, they observed spherical nanoparticles connected by dendritic structures that form due to turbulent mixing in some areas (Guo et al., 2023).

Furthermore, microfluidics offers outstanding opportunities for the nanodrug delivery systems production processes as it enhances controllability and uniformity (Zhang et al., 2023). In spite of the remarkable progress made in the creation and assessment of these systems through microfluidics, the shift of this innovative technology into actual industrial applications remains a hurdle. Achieving a kilogram production output or beyond each day is essential for both clinical investigations and large-scale manufacturing (Liu et al., 2018). However, the daily microfluidic-assisted-nanoparticle-production rate is usually in the milligram range (Kim et al., 2012). In order to address this issue, a nanoparticle production rate up to 3 kg/day was achieved by developing a coaxial turbulent jet mixer, which is suitable for industrial-scale production of nanodrug delivery systems (Lim et al., 2014). Other explored approaches are incrementing the channel dimensions (Gomez et al., 2014), and microfluidic channel parallelization (Shepherd et al., 2021). In this context, using an immobilized liquid lubricant perfluorodecalin layer was proposed by (Hwang et al., 2025) to prevent RNA-loaded lipid nanoparticles fouling. This technology was applied to a staggered herringbone microfluidic (SHM) mixing chip and reached more than 3 h of stable operation. Furthermore, they demonstrated this strategy’s compatibility with a parallelized microfluidic platform that gathers 256 SHM mixers, which assures stable production at L/h production rates suitable for commercial-scale applications.

### 3.2 Lab-on-chip tools for modeling tumor complexity and response

Microfluidic devices have emerged as an efficient tool for modeling tumor and normal tissue microenvironments (Nasiri et al., 2025b). Their ability to replicate tissue physiology and integrate biomechanical factors such as extracellular matrix and fluid dynamics parameters as flow rate, pressure, viscosity, surface tension, shear stress, and wettability (Liu et al., 2021; Sunildutt et al., 2023), has allowed them to create platforms to mimic the heterogeneity and complex cell organization and study cancer cell treatments, tumor evolution, chemosensibility, metastasis, and cell migration (Dsouza et al., 2022; Nejati et al., 2025; Ding et al., 2025). This performance is further enhanced by nanomaterials, which mainly improve detection sensitivity and biocompatibility (Tian et al., 2025).

Building on these advances, organ-on-a-chip (Ooc) represents a next-generation approach, capable of recreating controlled micro- and nanoenvironments in real time (Dsouza et al., 2022). These advanced biomimetic systems combine two or more cell types, including those derived from patients (Liu et al., 2025; Nejati et al., 2025), with microfluidics to replicate tumor heterogeneity, vascular networks, and three-dimensional architecture, facilitating the evaluation of drugs (Ma C. et al., 2021; Tian et al., 2025; Ayuso et al., 2022). In recent years, Ooc platforms have been used to assess the anticancer activity and phytochemical toxicity, for instance, *Spatholobi Caulis* tannin in cervical cancer (Wang et al., 2018), cis-stilbene glycoside and emodin-8-O-β-D-glucoside from *Polygonum multiflorum* in liver cancer (Deng et al., 2025), and panaxatriol from *Panax ginseng* C.A. Mayer in lung adenocarcinoma (Nasiri et al., 2025a). Additionally, this technology enables the study of complementary TME such as cell morphology, inflammatory process, migration process, protein expression, enzymatic activity, and oxygen and nutrient supply (Farooqi, 2022; Feng et al., 2025; Boul et al., 2021).

The integration of nanotechnology further enhances the phytochemical bioactivity, which has been evaluated in Ooc platforms. For instance, (Sharifi et al., 2020), used a liver-chip model to evaluate and compare anticancer activity of thymoquinone from *Nigella sativa*, both in its free form and encapsulated in chitosan-based nanoparticles. The results emphasize the potential of these systems to improve the analysis of antimetastatic activity, proliferation, migration, and colonization of tumor cells when encapsulated phytochemicals are delivered in tumor microenvironments.

Specialized Ooc platforms have been developed to address specific cancer-related processes. Vascular-on-a-chip models mimic angiogenesis and vascular responses; (Fayazbakhsh et al., 2023); used such a system to mimic blood vessels structure in angiogenesis process and assess the antioxidant effects of resveratrol-loaded gold nanoparticles on human umbilical vein endothelial cells under hyperglycemic conditions. The system enabled precise collagen level modulation and reactive oxygen species (ROS) real-time monitoring, revealing their reduction. BBB-on-a-chip system replicates central nervous system barriers to test drug delivery to brain tumors and evaluate parameters such as homeostasis and permeability. For instance, (Shi et al., 2023), developed a BBB-on-a-chip model using microvascular endothelial cells, pericytes, and mast cells to mimic the glioma microenvironment, enabling synergistic effect evaluation from traditional Chinese medicine phytochemicals to improve drug delivery and efficacy (Garcia et al., 2023) created a model combining 3 cell types: brain cells, human astrocytes-hippocampal (Ha-h), and Human brain vascular pericytes (HBVP), to test permeability and internalization of PLGA-encapsulated ferulic acid. Results showed improved internalization of this hydrophobic antioxidant, reduced ROS levels, and suggested nanoparticle size influences BBB permeability.

In addition, tumor-on-a-chip integrates tumor and stromal cells instead of healthy tissue cells, to mimic invasive tumor behaviour and TME dynamics (Li et al., 2023a; Tian et al., 2022), enabling a close evaluation of the anticancer potential of phytochemical extracts under conditions compared to 2D cultures. (Farooqi et al., 2022). used this model to evaluate anticancer activity of *A. cappadocicum* methanolic extract as well as to study ROS real-time monitoring, superoxide dismutase activity, and tumor biomarkers (e.g., urea, albumin) in the liver. Similarly, (Martins et al., 2023), designed a single-channel microfluidic devices with human glioblastoma cells to test the efficacy of free and nanoparticle-encapsulated docetaxel, reporting up to 50-fold lower IC_50_ values compared to conventional 2D monolayers, indicating higher tumor susceptibility under microfluidic conditions. Finally, spheroid on a chip combines multicellular tumor spheroids with microfluidics to assess long-term drug responses under perfusion, allowing compression of *in vivo* tumorigenesis and processes such as apoptosis and cell viability (Uzabakiriho et al., 2025; Nashimoto et al., 2020). For instance, a model with endothelial cells revealed that perfusion modulates paclitaxel sensitivity, underscored the role of stromal cells in angiogenesis, and identified flow rate as a determinant of drug efficacy and therapeutic response (Nashimoto et al., 2020).

Altogether, Ooc platforms provide a versatile and physiologically relevant technology for studying anticancer phytochemicals, nanoparticle-based therapies, tumor heterogeneity, and TME processes, bridging the gap between conventional 2D *in vitro* assays and *in vivo* models.

## 4 Conclusion

Phytochemical-based nanosystems enhance tumor-targeted therapy through multi-pathway modulation, improved bioavailability, stability, sustained and controlled release and efficacy. Among the different approaches, liposomes appear to be the most promising nanosystems in phytochemical delivery due to their properties such as: functional surface, low toxicity and minimal impact on healthy tissues. In this context, microfluidics enables the synthesis of precise-controlled size nanocarriers with potent activity for both laboratory research and industry settings. Although large-scale manufacturing remains challenging, parallelization emerged as a key strategy to scale production from milliliters to liters per hour. Lab-on-a-chip platforms complement microfluidic synthesis by providing biomimetic evaluation of transport, penetration, and response, facilitating patient-derived assays, increasing fidelity, lowering costs, and enhancing translational potential. Although several phytochemical nanomedicines have achieved FDA approval, microfluidic applications remain preclinical; nevertheless, ongoing innovation and patent activity highlight their promise for clinical translation.
